# Patient, physician, and caregiver perspectives on ovarian cancer treatment decision making: lessons from a qualitative pilot study

**DOI:** 10.1186/s40814-018-0283-7

**Published:** 2018-07-04

**Authors:** Rachel Pozzar, Laura-Mae Baldwin, Barbara A. Goff, Donna L. Berry

**Affiliations:** 1 School of Nursing, Bouvé College of Health Sciences, Northeastern University, 360 Huntington Avenue, Boston, MA 02115 USA; 2 Department of Family Medicine, University of Washington, Seattle, WA 98195 USA; 3 Department of Obstetrics & Gynecology, University of Washington, Seattle, WA 98195-6460 USA; 4 Department of Biobehavioral Nursing and Health Informatics, University of Washington, Seattle, WA 98195-7266 USA; 5 Department of Medicine, Dana-Farber Cancer Institute, Boston, MA 02215 USA

**Keywords:** Qualitative research, Ovarian cancer, Decision making, Caregivers

## Abstract

**Background:**

Ovarian cancer is the deadliest gynecologic malignancy and the fifth leading cause of cancer death among women living in the USA. Treatment for ovarian cancer that follows the guidelines published by the National Comprehensive Cancer Network is associated with a 33% decrease in disease-specific mortality, yet fewer than 40% of women with ovarian cancer receive guideline-adherent treatment. Little is known about the process by which women with ovarian cancer, their unpaid caregivers, and physicians make decisions about ovarian cancer treatment. We are planning to conduct a population-based study examining the ovarian cancer treatment decision-making process from the perspective of women with ovarian cancer, their caregivers, and physicians using a qualitative approach. Prior to embarking on a large-scale study, we determined it would be beneficial to pilot test our unpaid caregiver recruitment protocol and identify preliminary topics for the main study’s interview guide.

**Methods:**

We conducted a cross-sectional descriptive study using a qualitative approach. Data were collected via unstructured, individual interviews. Data were analyzed using modified grounded theory methods.

**Results:**

We interviewed six women with ovarian cancer, four unpaid caregivers, and three physicians. The recruitment protocol successfully recruited patient participants but did not allow for direct recruitment of unpaid caregivers, which presented logistical difficulties. The interview guide was adequate to elicit participants’ discussion of the major topics of interest; however, the opening statement needed modification to account for physician participants’ specialties. Patient and caregiver participants identified three major categories of concepts describing the process of ovarian cancer treatment decision making: (a) choosing a provider, (b) choosing a facility, and (c) choosing a treatment. All three groups of participants addressed the influence of geographic location on treatment decisions, while physicians described encounters with patients declining recommended treatment.

**Conclusions:**

This pilot study met our objectives of testing unpaid caregiver recruitment procedures and identifying topics to include in the interview guide for a planned grounded theory study. Although the thematic results of this study are preliminary, the categories of concepts described by participants provide a framework for the exploration of patient, unpaid caregiver, and physician perspectives of ovarian cancer treatment decision making.

## Backgound

Ovarian cancer is the leading cause of death from gynecologic cancer and the fifth leading cause of cancer death among women living in the USA [1]. Sixty-five percent of women with ovarian cancer have advanced disease at the time of diagnosis, at which point the 5-year relative survival rate ranges from 17% for stage IV disease to 39% for stage III disease [1, 2]. Treatment of ovarian cancer often requires extensive surgery and complex decisions regarding pre- or postoperative chemotherapy. Women with a new diagnosis of ovarian cancer who are treated according to the guidelines issued by the National Comprehensive Cancer Network (NCCN) experience a 33% decrease in disease-specific mortality compared to those who are not; yet nationwide, fewer than 40% of women with ovarian cancer receive NCCN guideline-adherent treatment [3].

The reasons that so few women with ovarian cancer receive the standard of care are largely unknown. In a recent systematic review, Pozzar and Berry [4] found that while age, race, ethnicity, socioeconomic status, disease stage, and hospital case volume are the factors most commonly associated with receipt of guideline-based treatment, the mechanisms by which these factors influence treatment selection is unclear. In addition, although consultation with a gynecologic oncologist is positively associated with the receipt of guideline-based care [5, 6], a recent population-based study by Warren and colleagues [7] revealed that the majority of women who consult with a gynecologic oncologist still do not receive the standard of care. It is likely that patients, physicians, and caregivers all play a role in the ovarian cancer treatment decision-making process. Treatment decision research for other malignancies has shown that patients’ beliefs and preferences play as significant a role as medical issues in determining type of treatment received [8–12]. Physician factors such as beliefs about decision-making roles, power dynamics, and communication style have also been found to influence cancer treatment decisions [13]. With regard to ovarian cancer, a systematic literature review conducted by Luketina et al. [14] found that the physician-patient relationship influences treatment decision making, yet no known study has explored this process from the physician’s perspective. Similarly, the role of unpaid caregivers in supporting cancer treatment decision making has been documented [15–17]; however, no known studies have explored the topic of ovarian cancer treatment decision making from the caregiver’s perspective.

Improved understanding of the process by which women with ovarian cancer, their physicians, and their unpaid caregivers make treatment decisions may inform interventions to promote informed decision making. In turn, these interventions have the potential to increase the proportion of women with ovarian cancer who receive guideline-based care. Given the limited literature on this topic, we are planning to conduct a population-based qualitative study examining the ovarian cancer treatment decision-making process from the perspective of women with ovarian cancer, their caregivers, and physicians using grounded theory. Grounded theory is a systematic research method that focuses on a process that occurs over time [18]. In a grounded theory study, the researcher uses inductive reasoning to develop categories comprised of concepts that are uncovered during data analysis. Concepts, categories, and their relationships with one another are refined in an iterative process known as *constant comparison* [19]. Data collection and analysis take place concurrently, with the researcher purposively recruiting participants who may be able to provide an alternate perspective of emerging concepts. The product of a fully realized grounded theory study is a theory of a process or action that is “grounded in the data” [18, 19]. Prior to embarking on a large-scale grounded theory study, we determined it would be beneficial to pilot test our unpaid caregiver recruitment and interview protocols.

Qualitative pilot studies are not conducted routinely, and authors such as Morse [20] have cautioned against drawing conclusions from incomplete data. Corbin and Strauss [19] affirmed that a pilot study is not typically a prerequisite to a grounded theory study; however, they acknowledged that in certain situations, a pilot study may help the investigator to refine the problem area. In addition, Creswell [18] noted that a qualitative pilot study may be used to trial and improve upon recruitment procedures and interview protocols. Considering these views, we concluded that a pilot study would permit us to make judicious use of limited resources by identifying potential flaws in our protocols prior to engaging in large-scale recruitment. In addition, we sought to ensure that no undue burden would be imposed on individuals who volunteered to participate in the study and aimed to identify topics that were difficult for participants to address.

The purpose of this study was to pilot test the procedures for recruiting unpaid caregivers and the interview protocols of a planned grounded theory study of the ovarian cancer treatment decision-making process. In addition, we sought to generate a preliminary list of topics for the interview guide of the larger study. This article is a report of our experiences conducting a pilot study of patient, physician, and caregiver perspectives on ovarian cancer treatment decision making; our lessons learned; and our preliminary findings.

## Methods

### Study design

We conducted a cross-sectional, descriptive study using a qualitative approach. We modified the grounded theory method described by Corbin and Strauss [19] to fit the scope and purpose of our pilot study. Specifically, we (a) limited data analysis to open coding, (b) utilized a convenience rather than purposive sample, and (c) halted recruitment when we had learned consistent lessons from the protocol, as opposed to stopping at a saturation point for concepts and process as in a fully realized grounded theory study.

### Participants and setting

Patient participants were recruited from a gynecologic oncology service within a National Cancer Institute (NCI)-designated cancer center located in the Pacific Northwestern United States. Unpaid caregiver participants were identified and invited by patient participants. Physician participants were recruited from the same institution as patient participants. Eligible patients were adult, English-speaking women with histologically-proven ovarian cancer of any stage who had been newly diagnosed within the last 12 months. Unpaid caregivers were eligible if English-speaking. Physicians were eligible if they were known to provide care to women with a diagnosis of ovarian cancer at the recruitment site. We planned to recruit no more than six women with ovarian cancer, six unpaid caregivers, and six physicians.

### Procedure

#### Recruitment

The study was approved by the Fred Hutchinson Cancer Research Center Institutional Review Board (IRB) which issued a waiver for written consent. The Northeastern University IRB approved author RP’s participation in data analysis. The principal investigator (BG) invited eight patients and five physicians to learn more about the study, then provided the contact information of interested potential participants to the co-investigator (DB) for follow-up. No more than two telephone contacts were attempted to recruit participants. Once contact with the co-investigator was made, a study information sheet was mailed or emailed to each potential participant and an appointment was made for a telephone interview. At the beginning of each interview appointment, the co-investigator discussed the potential risks and benefits of study participation with participants and provided an opportunity to ask questions. The optional nature of the study was emphasized. Interviews began after verbal informed consent was obtained.

At the end of each patient interview, participants were asked whether an unpaid caregiver was involved in their care. If a caregiver was involved, the co-investigator asked the participant to tell the caregiver about the study and ask the caregiver to contact the co-investigator. No direct recruitment was conducted until unpaid caregivers had made an initial attempt to contact the co-investigator.

#### Interviews

Individual, unstructured interviews were conducted over the telephone by the co-investigator. The opening query for each patient interview was “Please discuss your cancer treatment decision, including all important issues and concerns and how you made your decision; including the who, what, where, and when of your decision.” Participants were prompted, only if needed, to address the following topics: communication with healthcare providers, transportation to care facilities, issues related to work and family roles, age and education, symptoms, partner/sexual issues, and survival. Demographic information was collected at the end of each patient interview.

Interviews with unpaid caregivers were opened with the same question and proceeded with similar prompts as with patient participants, with slight word changes and prompts tailored to the caregiver role. Interviews with physicians were conducted similarly to interviews with patients and caregivers and used similar prompts. The opening query for physician interviews was “Please discuss the ovarian cancer treatment decision process with a typical patient.” To reduce participant burden, demographic data were not collected from unpaid caregiver and physician participants. All interviews were audio recorded, processed to remove all protected health information, and transcribed verbatim.

### Data analysis

All de-identified transcripts were entered into NVivo 11 Pro [21] for analysis. Each interview transcript was read in its entirety by two authors (DB and RP). On the second read-through, RP used open, line-by-line coding to categorize and label the raw data. Codes within and between transcripts were assigned using the constant comparative method [19]. Definitions, interpretations, and areas in need of further exploration were documented in analytic memos. On completion of open coding, codes were reviewed for redundancies and refined. Codes were then grouped into categories when possible. Beginning evidence of conceptual and temporal relationships between concepts was documented using diagrams. To enhance validation of findings, DB reviewed all coding and analytic memos. A methods memo was created and edited during the study to document recruitment and interview protocol issues.

## Results

Six out of eight potential patient participants were interviewed. Seven patients were contacted and agreed to participate (88%); however, one reported that she was “too sick” to participate in the study on the day of the interview appointment. One patient could not be reached after two telephone calls. Six patient participants identified an unpaid caregiver; of these six, four (67%) followed up with the co-investigator for an interview. Three out of five physicians (60%) agreed to an interview.

All six patient participants were self-identified as white, non-Hispanic. Two of six were between 50 and 59 years old, two between 60 and 69 years old, and two 70 years old or older. Five of six were married or partnered and one was widowed. Four of six had at least some college education, one was a high school graduate, and one did not answer. Most (5 of 6) were retired, and one was working full-time for pay. Four of six reported an annual household income equal to or greater than $60,000, while two declined to answer. Half were insured by Medicare, two were privately insured, and one was insured by both Medicare and private insurance. All patient participants reported having been diagnosed within the past year. All were undergoing primary treatment, with most (4 of 6) having received both surgery and at least 1 cycle of chemotherapy at the time of the interview. During the interview process, caregiver participants described themselves as a partner, daughter, niece, or cousin of the patient participant. Physician participants described themselves as a general surgeon, gynecologist, or gynecologic oncologist.

### Interview protocol

The preliminary results confirmed the appropriateness of the interview prompts and identified categories of issues encountered during treatment decision making. Two out of three physician participants, a general surgeon and a gynecologist, required additional clarification about the study topic following the opening statement. In both cases, the physicians explained that their role was to refer patients to gynecologic oncology for ovarian cancer diagnosis and treatment. During these interviews, the interviewer clarified the opening statement to “Please explain how you help a patient during the process of referral to gynecologic oncology.”

Patient and caregiver participants spontaneously addressed most topics in the interview guide and required no clarification of the study topic. Without prompting, patient participants responded to the opening question by telling their story beginning with the circumstances of their diagnosis and the process by which they selected a gynecologic oncologist. No topics or prompts were distressing to participants. Telephone interviews were conducted and recorded without difficulty. Each interview lasted approximately 60 min.

### Preliminary themes

Three major categories of concepts describing the process of ovarian cancer treatment decision making were documented during this analysis: (a) *choosing a provider*, (b) *choosing a facility*, and (c) *choosing a treatment*. What follows is a summary of each category and its relevant concepts.

#### Choosing a provider

In most cases, patient participants began the response to the opening query by describing the process by which they selected a gynecologic oncologist. Patient participants described being referred to a gynecologic oncologist by their gastroenterologist, gynecologist, general surgeon, or primary care physician.

Four out of six patients were referred initially to their chosen gynecologic oncologist, while two had an initial consultation with the gynecologic oncologist to whom they were referred, but chose to transfer their care to a physician from whom they received a second opinion. All patient participants met with the gynecologic oncologist to whom they were initially referred. Among patient participants who sought a second opinion, one described being satisfied with her initial consultation but wanting to explore her options given her assessment of the magnitude of her decision:

We went to the appointment and, you know, it was a good appointment. …after going home and you know — thinking about it for a couple of days trying to figure it all out, we thought, well, it’s a huge decision, so we should go get a second opinion. (PT5)

The other patient participant who sought a second opinion described a negative interaction with an individual who worked with the gynecologic oncologist to whom she was initially referred. This experience prompted her to seek a second consultation elsewhere.

Patient participants described several factors that influenced their eventual choice of gynecologic oncologist. Most commonly, these included the physician’s reputation, the physician’s bedside manner, and the recommendations of family and friends. One of the participants who received a second opinion described selecting her gynecologic oncologist based on the specific treatment the physician proposed. This participant reported having read about a potential survival benefit associated with intraperitoneal chemotherapy and decided to receive care from the physician who offered this treatment.

Unpaid caregivers described playing a primarily supportive role in the provider selection process. One caregiver described sharing the patient’s dissatisfaction with the gynecologic oncologist to whom she was initially referred and encouraging the patient to pursue a second opinion. Caregivers were similar to patients in that they valued provider reputation and interpersonal characteristics.

The general surgeon and gynecologist described assisting patients suspected of having ovarian cancer to find a gynecologic oncologist for evaluation and treatment. The gynecologic oncologist described caring for patients who were referred to her but lived a long distance from her practice site, some of whom had to travel by air. In these cases, the gynecologic oncologist described having to balance patients’ transportation concerns with the clinical necessity of follow-up appointments. Some long-distance patients ultimately elected to receive treatment closer to home. In these cases, the gynecologic oncologist sought to identify specialists in patients’ local communities who were prepared to assume care.

#### Choosing a facility

As when choosing a provider, patient participants in this sample often considered facility reputation when making decisions about where to receive treatment. One patient (PT5) described her facility of choice as doing a lot of “research and trials” and having “more resources,” while another (PT3) cited her facility’s affiliation with an NCI-designated cancer center in the region. Not all patients researched facilities prior to selecting one. For example, one participant explained:

…after I said we’d come, then I think I started looking and thought, ‘Well, this is really a good choice because this is a well-recognized cancer treatment center.’ (PT1)

Facility location was commonly addressed by patient participants. Five out of six patient participants described traveling at least 25 miles (approximately 40 km) to receive treatment at their chosen facility. Patients cited a perceived lack of local specialists as one reason they were willing to travel outside of their area of residence:

My health is worth, you know, ninety miles [approximately 145 kilometers] a few times a month. …I have the best [doctors] down there. Ninety miles, ninety miles is nothing. (PT2)

One participant described her decision to relocate to a different state:

I would have had to drive forty-five miles [approximately 72 kilometers] over a mountain pass to another town to see an oncologist. And there’s no oncologist in the town where I live. …There would have been no surgical oncologists, obviously. And both places just have, um, general surgeons. (PT1)

To reduce travel burden, three out of six patient participants considered undergoing surgery at one treatment facility but receiving chemotherapy or radiation at a facility closer to home. Ultimately, one participant decided against this option while two decided in favor of it. For some participants who regularly traveled far from home to receive treatment, it was important to have the support of caregivers living near the treatment facility. Physician participants similarly described transportation as a barrier to their patients receiving treatment at the cancer center. Physicians acknowledged the importance of social service agencies in arranging transportation for patients with limited financial resources.

#### Choosing a treatment

Patient participants varied in the extent to which they perceived they had a choice to make about treatment. Three out of six patients referred to the importance of moving quickly to start treatment. One insinuated that time pressure had a detrimental effect on decision quality:

At least my initial reaction was kind of like a panic. It’s like, ‘We’ve got to go. We’ve got to get this cut out now, and, you know, we’ve got to get it done within the next three days.’ …And how [do] you slow that down and get more information to process… …And how to help those people make good choices—not in a panic kind of good choices. (PT1)

Several patient participants described playing a passive role in the treatment decision-making process. Among these participants, trust in the physician was a common theme.

I thought she [the gynecologic oncologist] was extremely knowledgeable. And so, you know, it’s just the level of trust and her saying, ‘This is the way to proceed.’ (PT1)

Each of the three unpaid caregivers who specifically discussed the process of selecting a treatment attributed the decision to the physician. Here, too, a sense of urgency to start treatment played a role in the caregiver’s willingness to follow the physician’s advice.

This is what they’re [the physicians are] saying; we’re going to do it. And really not even any other thoughts of more opinions, because the cancer markers were so high… (CG4)

Two patient participants described making a specific treatment decision. Both framed the decision as a process in which they and their family participated. None of the patient participants described seriously considering the option of “no treatment.” Patients viewed the alternative to treatment and its side effects as death; therefore, they viewed their choice to pursue treatment as a choice to live.

In contrast, all three physician participants had cared for patients who declined to pursue treatment. In addition, physicians described experiences with patients who preferred to pursue complementary and alternative therapies in lieu of recommended treatment. Physicians perceived that patients who voiced these preferences were generally well educated but mistrustful of the health care system. When caring for patients who expressed a desire to delay or forgo recommended treatment in favor of complementary or alternative therapies, physicians struggled to balance respect for patient autonomy with the desire to do no harm. One physician participant reported that in some cases, the patient’s desire to maintain control over treatment decisions promoted physician-patient interactions that more closely resembled bargaining than shared decision making.

## Discussion

We accomplished our goal of testing the unpaid caregiver recruitment procedures and interview protocols of a planned grounded theory study. The findings of this pilot study suggest that women make three broad and interrelated decisions after being diagnosed with ovarian cancer: (a) *choosing a provider*, (b) *choosing a facility*, and (c) *choosing a treatment*. The major concepts identified during this pilot study will be included as prompts in the larger study’s interview guide. Specifically, patient and caregiver participants will be prompted about institution and provider reputation, while all participants will be prompted about the consideration of complementary and alternative therapies.

Patient participants described the influence of physician referrals, personal recommendations, and physician interpersonal characteristics and reputation on their choice of provider. These findings highlight the importance of exploring the process by which women with ovarian cancer select a provider in the main study. Because this is a pilot study, these findings are preliminary and should be interpreted with caution. The patient participants in this study were recruited from a region of the country that is served by an NCI-designated cancer center. The factors that influence provider selection may well differ among a larger, more diverse sample of women, some of whom may not have access to a similar facility. Likewise, the finding that generalist physicians initiate the referral process to a gynecologic oncologist may be specific to the setting of this pilot study: nationwide, approximately one quarter of women with ovarian cancer do not consult with a gynecologic oncologist [7]. In the planned study, purposive sampling will permit recruitment of patient participants who do not consult a gynecologic oncologist but are managed by gynecologists, general surgeons, and medical oncologists instead.

When choosing a facility, the patient participants in this sample considered an institution’s reputation, distance from home, and proximity to supportive caregivers. Physicians described challenges faced by women who lack transportation or must travel long distances to reach the treatment center. These findings provide context for what is known from the quantitative literature. In a study of factors associated with ovarian cancer treatment that adheres to NCCN guidelines, Bristow et al. [22] found that travel distance between 20 and 50 miles (approximately 32 and 80 km, respectively) was significantly associated with the receipt of guideline-based care, while travel distance over 50 miles was associated with deviation from guidelines. Access to transportation and ability to travel may therefore have an impact on quality of care and treatment outcomes.

Only two of six patients described selecting a specific treatment option. This finding is congruent with previous findings in the literature that suggest the extent to which women with ovarian cancer participate in treatment decision making varies [23, 24]. Patients in the current study reported that the urgency with which they started treatment limited the extent to which they participated in shared decision making. This may explain why trust in one’s physician was a common theme described by the patients in this study. Prior studies of women with ovarian cancer have found that feeling overwhelmed at the time of diagnosis served as a barrier to participation in treatment decision making [25–28]. Additional literature suggests that cancer patients’ preferred involvement in shared decision making may change as they proceed along the continuum of care [29, 30]. This is an area in need of further study given the evidence that women with ovarian cancer believe it is important to participate in the treatment decision-making process [23, 31–33] and that participation in treatment decision making is associated with improved quality of life among ovarian cancer survivors [23]. Although the patient participants in this pilot study were relatively homogenous in terms of time since diagnosis, the use of purposive sampling in the main study will allow for recruitment of participants in different phases of care.

The patient participants in this pilot study described a desire to receive the most effective treatment, a finding that reflects what has been previously reported in the literature [27, 33–35]. In the main study, it will be important to explore this finding among participants whose socioeconomic and educational backgrounds differ from those in the current sample, comprising primarily affluent patient participants. Likewise, the results of this pilot suggest that a subset of women with ovarian cancer may prefer to pursue complementary and alternative therapies in lieu of recommended treatment. Although prior research has examined factors associated with forgoing recommended chemotherapy [36], it is likely that the decision to pursue complementary and alternative therapies represents a separate phenomenon. The use of complementary and alternative therapies in conjunction with conventional medicine is common among women with gynecologic malignancies [37]; however, few studies have sought to explore the process by which an individual with cancer decides to pursue complementary and alternative therapies in lieu of conventional medicine [38]. Further research is warranted to explore and expand this finding, particularly in a larger and more diverse sample.

### Practical implications

One lesson learned from this pilot study was to tailor the physician interview based on specialty. The general surgeon and gynecologist participants had difficulty commenting on their approach to patients newly diagnosed with ovarian cancer because at this facility, referral to a gynecologic oncologist typically preceded diagnosis. Modification of the opening statement to inquire about either the physicians’ approach to the treatment decision-making process or to referral allowed interviews to proceed without further difficulty. Importantly, this pilot study was conducted in a setting in which gynecologic oncologists were available locally. In settings with fewer available gynecologic oncologists, the role of other providers such as gynecologists and general surgeons may differ. It will be important in our population-based study to ensure the interview guide accounts for the variety of roles that general surgeons, gynecologists, and other oncology providers may play in treating and referring women with suspected or confirmed ovarian cancer.

Recruitment of unpaid caregivers was a challenge in this study because the recruitment protocol did not allow for direct recruitment of unpaid caregivers; rather, we relied upon patient participants to ask their caregivers to contact us to discuss the study. For our larger study, we plan to modify our study protocol and IRB proposal to allow for direct contact of unpaid caregivers rather than relying upon patient participants to facilitate the first contact.

### Limitations

The thematic results of this pilot study should be interpreted with caution for several reasons. First, patient recruitment was limited to one surgical service at a comprehensive cancer center in the Pacific Northwest. It will be important in our population-based study to explore the perspectives of women who do not receive care at a comprehensive cancer center. Similarly, physician participants were limited to the colleagues of one investigator at the recruitment site. The resultant patient and physician samples were small and relatively homogenous, precluding the use of triangulation and negative case analysis to validate interpretation of findings [18]. Most notably, patient participants were primarily well-educated, affluent, white, and non-Hispanic. For our larger study, we plan to recruit diverse patient and physician participants through national cancer registries [39]. Consistent with grounded theory methods, we plan to use purposive sampling to solicit a variety of perspectives on the concepts identified both during this pilot and during subsequent analyses.

The second major limitation of the current study was that, by design, the amount of data collected was not adequate to achieve theoretical saturation and analysis was limited to open coding [19]. Therefore, the thematic results of this study should be viewed as preliminary and incomplete.

## Conclusion

This pilot study met our objectives of testing the unpaid caregiver recruitment procedures and interview protocols of a planned grounded theory study of ovarian cancer treatment decision making. Our experiences conducting this pilot study will inform our approach to recruitment in the future, particularly the recruitment of unpaid caregivers. The preliminary thematic results of this pilot study provided insight into the major themes of ovarian cancer treatment decision making and allowed us to refine our list of prompts in the interview guide for the larger study. Given the paucity of literature describing cancer treatment decision making from the perspective of patients, caregivers, and physicians, the results of this pilot study may be of interest to researchers planning to conduct similar investigations.

## Abbreviations

IRB: Institutional Review Board
NCCN: National Comprehensive Cancer Network
NCI: National Cancer Institute
SEER: Surveillance, Epidemiology, and End Results
